# Paro Declaration: commitment to addressing mental health challenges in the South-East Asia Region

**DOI:** 10.2471/BLT.23.290671

**Published:** 2023-09-14

**Authors:** Dechen Wangmo, Zahid Maleque, Choe Kyong Chol, Mansukh Mandaviya, Budi Gunadi Sadikin, Ahmed Naseem, Mohan Bahadur Basnet, Keheliya Rambukwella, Sathit Pitutecha, Élia A A dos Reis Amaral, Tedros Adhanom Ghebreyesus, Poonam Khetrapal Singh

**Affiliations:** a Ministry of Health, Thimphu, Bhutan.; b Ministry of Health and Family Welfare, Dhaka, Bangladesh.; c Ministry of Public Health, Pyongyang, Democratic People’s Republic of Korea.; d Ministry of Health and Family Welfare, New Delhi, India.; e Ministry of Health, Jakarta, Indonesia.; f Ministry of Health, Malé, Maldives.; g Ministry of Health and Population, Kathmandu, Nepal.; h Ministry of Health, Colombo, Sri Lanka.; i Ministry of Public Health, Bangkok, Thailand.; j Ministry of Health, Dili, Timor-Leste.; k World Health Organization, Geneva, Switzerland.; l World Health Organization Regional Office for South-East Asia, Indraprastha Estate, Mahatma Gandhi Marg, New Delhi, 110 002, India.

The World Health Organization (WHO) defines mental health as “a state of mental well-being that enables people to cope with the stresses of life, to realize their abilities, to learn well and work well, and to contribute to their communities.”^1^ Mental disorders have remained among the top 10 leading causes of disease burden worldwide, with no reducing trend since 1990.^2^ Almost 1 billion people live with different mental health conditions around the world, of whom 260 million people are from the WHO South-East Asia Region.^2^ Globally, mental health conditions contribute the most to years of healthy life lost to disability, with depression being the largest contributor and schizophrenia the single-most disabling condition.^3^ In 2019, an estimated 703 000 people across all ages lost their life to suicide, which accounts for 1 in 100 deaths globally; 200 000 of these deaths occur in the WHO South-East Asia Region every year.^4^^,^^5^ People with severe mental disorders die 10 to 20 years earlier than the general population^6^ and excess deaths in these patients are attributable to comorbidities^7^ and alcohol use disorders.^8^

Investment in mental health remains very low across the Region and is below 1 United States dollar (US$) per capita in several countries.^4^ The World Economic Forum has calculated that in 2010, a broadly defined set of mental health conditions cost the world’s economy approximately US$ 2.5 trillion, which by 2030 is expected to rise to US$ 6 trillion.^9^ At the same time, evidence shows that investing just US$ 1 in scaled-up treatment for priority mental health conditions such as depression and anxiety yields a return of US$ 5 in improved health and productivity.^10^

Inadequate investment in mental health systems, services and health workforce has resulted in large treatment gaps in low-income countries,^11^ including those of the WHO South-East Asia Region, such as Bangladesh and India.^12^^,^^13^ The inadequate number of health workers for mental health hampers effective delivery of services. Investments specifically aimed at improving services through primary care are lacking, with many countries focusing on specialized centres for mental health in cities. The paucity of up-to-date data on mental health impedes informed policy-making. Most countries lack national-level programmes to address stigma and improve mental health literacy.

The coronavirus disease 2019 (COVID-19) pandemic exacerbated mental health risks and exposed vulnerabilities aggravated by neglect of mental health over the years, and underscored the critical importance of mental health for overall health and well-being. Globally, in the first year of the pandemic, an estimated 25% rise in cases of anxiety and depressive disorders was reported.^14^ The pandemic has considerably affected the mental health of young people and women.^4^ Loneliness, fear of infection, suffering and death, grief after bereavement and financial worries have all been cited as stressors leading to anxiety and depression. Effective community-based and community-led psychological interventions, including innovative and technology-based service delivery, helped mitigate mental health consequences during the pandemic; these interventions need to be upscaled.^15^

In 2022, the Paro Ministerial Declaration was unanimously adopted at the 75th WHO Regional Committee of South-East Asia in Paro, Bhutan, following the Ministerial Roundtable on addressing mental health through primary care and community engagement.^16^

The Declaration calls for several transformative changes. One transformation is moving away from the current disease-centred approach towards a well-being focus. This change requires a shift from the predominant emphasis on diagnosing and treating mental disorders after they have developed, to an approach that places people at the centre of interventions, prioritizes lifestyle and behavioural factors and early interventions, and addresses known determinants of mental health. The Declaration also calls for community engagement, which involves creating opportunities for individuals and communities to actively participate in mental health initiatives, support and advocacy. Engaging communities can help reduce stigma, increase awareness and improve access to mental health services. Another transformation called for in the Declaration is shifting from a purely biomedical approach to a bio-psychosocial approach that involves a fundamental change in how mental health is understood, assessed and treated. This approach recognizes that mental health is influenced not only by biological factors but also by psychological and social factors. 

The Declaration also calls for moving from tertiary care to community care, and from specialist to non-specialist cadres to ensure that mental health is an integral component of universal health coverage.

The Declaration urges Member States in the WHO South-East Asia Region to develop and implement multisectoral policies across the life-course to address mental health risks, and reduce treatment gaps to ensure that mental health services reach all those in need, close to where they live, without leading to financial hardship.

To translate the Paro Declaration into practice, the Mental Health Action Plan for WHO South-East Asia Region^17^ was consultatively produced in collaboration with Member States. The plan includes key areas for implementation as well as indicators for tracking progress. The plan focuses on decentralization of services and strengthening of community-based mental health networks.

To support the decentralization process, a toolkit for expanding community mental health services is being developed with inputs from countries. Currently, district-level mental health services are being strengthened through the WHO Special Initiative for Mental Health^18^ in Bangladesh and Nepal, which has increased access to mental health services through primary care.

WHO remains committed to increase investments in mental health services, primary care and health workforce capacities as a key strategy to reduce the prevention and treatment gaps, through whole-of-society and whole-of-government approaches and multistakeholder initiatives. We will actively implement the provisions of the Declaration and deliver outcomes. The Regional Committee also made the decision to follow up progress on implementation of this Declaration every two years to ensure accountability.

Improving the mental health of populations leads to increased economic productivity and greater social development. The need for holistic mental health interventions is vital to taking the human development agenda forward. The WHO Regional Office for South-East Asia has worked continuously to assist Member States, which has resulted in many countries adopting mental health policies, plans and laws for advancing the mental health of their people. Yet, more needs to be done. The 2022 *World mental health report: transforming mental health for all* highlights the transformations required to address these challenges.^4^ The Paro Ministerial Declaration adopted on 6 September 2022 is therefore timely, and a major milestone in accelerating progress on mental health and well-being within Member States of the WHO South-East Asia Region.
